# A carefully planned resistance training program improves strength, fitness and depressive symptoms for a woman with type 2 diabetes

**DOI:** 10.1530/EDM-24-0091

**Published:** 2024-10-03

**Authors:** Mario I Hernandez, Ansley B Devine, Joseph Ramsey, Emily Dow, Carol S Johnston

**Affiliations:** 1 College of Health Solutions, Arizona State University, Phoenix Campus, Phoenix, Arizona, USA

**Keywords:** diabetes, resistance training, strength, depression, lipopolysaccharide

## Abstract

**Summary:**

Depression in type 2 diabetes (T2D) is estimated at 50% vs 18% among US adults, and markers of inflammation, which are tightly linked to hyperglycemia, are 5- to 50-fold higher in adults with T2D. Although lifestyle modifications are recommended for managing diabetes, resistance training (RT) is not commonly considered. This case report examined the practicality of implementing a structured RT protocol in a highly sedentary woman with T2D and depressive symptomology and assessed changes in strength, fitness, depression, and inflammation. The 59-year-old participant (body mass index: 38.1 kg/m^2^) was diagnosed in 2015. She had hypertension and bronchial asthma, was highly sedentary, and was clinically depressed based on validated measures: The Center for Epidemiological Studies Depression (CES-D) questionnaire and the Profile of Mood States (POMS) questionnaire. She had quit smoking 6 months earlier. The estimated 1RM guided the exercise prescription that used progressive overload to improve strength and promote the accretion of lean body mass. All exercise sessions (~45 minutes duration; 3× weekly) were supervised by trained personnel. After 8 weeks, total strength improved 135%. Heart rate was reduced by 14%, and depression symptomology fell into normal ranges. Although RT improved strength, fitness, and depressive symptomology, RT did not improve HbA1c, HOMA-IR, or inflammation emphasizing the need for a comprehensive treatment strategy. Simple assessments can be performed to determine the fitness and mental health of individuals with T2D, and incorporating an exercise prescription to standard care to address these key health determinants will empower patients to actively engage in their health care.

**Learning points:**

- A progressive, individualized resistance training program is feasible and improves muscular strength, fitness level, and mental health in a high-risk individual with type 2 diabetes and multiple comorbidities.
- Flexibility with resistance training prescription to adapt to patient’s needs and abilities contributes to exercise adherence and successful outcomes
- Physical activity assessment and exercise prescription should be a part of standard care for patients with diabetes.

## Background

Diabetes is often complicated by depressive symptomology and chronic inflammation. The prevalence of depression in type 2 diabetes (T2D) has been estimated at 50% compared to 18% among US adults (1), and an NHANES 2005–2018 cohort analysis reported that mortality risk was increased 20–67% in individuals living with diabetes (*n* = 5695) who experienced moderate-to-severe depression (2). Moreover, several of the most commonly prescribed medications for T2D, metformin and insulin, have been linked to depression, particularly at high dosages (3). Serum C-reactive protein (CRP) concentrations, a general test of inflammation, are 5- to 50-fold higher in adults with T2D compared to healthy adults (4). The metabolic inflammation that accompanies insulin resistance also contributes to the progression of hyperglycemia, the complications of diabetes, and depression. Antihyperglycemic medications have demonstrated anti-inflammatory properties (5); however, since this effect is likely linked to medication-induced improvements in insulin resistance and not to direct impacts on immune system activation, the inflammatory condition persists if hyperglycemia is present.

Dietary and lifestyle modifications are recommended for managing diabetes and offer a range of benefits without the side effects often linked to antihyperglycemic medications. For example, resistance training (RT) improves muscle strength and indicators of cardiovascular health in patients with T2D (6); yet, RT is not commonly recommended by practitioners. Furthermore, less is known regarding the impact of RT on systemic inflammation or depressive symptomology, particularly in patients with poor glucose control. This case report aimed to examine the practicality of implementing a structured RT protocol in a highly sedentary woman with T2D and persistent hyperglycemia and assess the potential benefits of RT for improving strength, depressive symptomology, and health indicators including fitness, depression scores, inflammation, and insulin resistance.

## Case presentation

The participant was a 59-year-old female. She was diagnosed with T2D in 2015. Additionally, she had hypertension and bronchial asthma (diagnosed in 2016). She was a former smoker and had quit 6 months earlier. Her medications were prescribed by a physician: dulaglutide (2022), losartan (2023), and conjugated estrogens vaginal cream (2013). At baseline, body weight and fat-free mass were 101 kg and 51 kg respectively, and the body mass index was 38.1 kg/m^2^ (Table 1). She took cetirizine regularly and consumed vitamin B12 and magnesium daily. Her physician had approved her participation in this investigation. The participant provided written consent, and the investigation was approved by the Institutional Review Board at Arizona State University. Additionally, the participant completed the consent form for publication in a Bioscientifica Journal.

**Table 1:** Change in measures following 8 weeks of supervised RT.

	Baseline	Week 8	% change
Height (cm)	162.9		–
Weight (kg)	101.0	103.7	+2.7
Fat-free mass (kg)	50.9	53.0	+4.1
Percent body fat (%)	49.6	48.9	–1.4
BMI (kg/m^2^)	38.1	39.1	+2.6
Total strength, kg	48.6	113.6	+134.7
Systolic blood pressure (mm Hg)	115.0	122.0	+6.1
Diastolic blood pressure (mm Hg)	70.0	75.0	+7.1
Resting heart rate (BPM)	81.0	70.0	–13.6
HbA1c (%)	8.4	9.0	+7.1
C-reactive protein (mg/L)	10.1	9.4	–6.9
Fasting glucose (mg/dL)	223.0	260.0	+16.6
Fasting insulin (µIU/mL)	69.0	76.0	+10.1
HOMA-IR	38.0	48.8	+28.4
LBP (μg/mL)	26.8	27.3	+1.9
C-reactive protein (mg/L)	10.1	9.4	–6.9
CES-D score	17	13	–24.0
POMS score	63	48	–23.8

## Investigation

The participant did not engage in physical activity (score = 6 on the Godin–Shephard Leisure Time Physical Activity Questionnaire; <14 indicates insufficiently active), and resting heart rate (81 bpm) suggested below average fitness level (Table 1). Depressive symptoms were assessed using widely applied validated questionnaires: The Center for Epidemiological Studies Depression (CES-D) questionnaire and the Profile of Mood States (POMS) questionnaire (7). The CES-D, which is recommended for use in patients with T2D (8), is a 20-question Likert scale tool that assesses how one has felt over the past week. Scores range from 0–60, and scores ≥16 indicate risk for clinical depression. The patient scored 17 at baseline indicating possible clinical depression (Table 1). The POMS assessment utilizes an index of 65 adjectives to describe the presence of six mood states (depression, tension, fatigue, confusion, and vigor) over the prior 7 days; higher scores correspond to greater mood disturbance, and, in women, scores >70 suggested clinical risk for depression, and a depression sub-score ≥7 can indicate major depression. The participant scored 63 and 8 respectively at baseline indicating a risk of depression. CRP concentrations were markedly increased at baseline (10.1 mg/L) indicating clinically significant inflammation (Table 1).

## Treatment

Prior to RT initiation, the 10-repetition maximum (RM) assessment was performed on eight different resistance machines that focused on major muscle groups and was converted to an estimated 1 RM using the Brzycki formula: the maximum weight lifted for 10 reps divided by (1.0278–0.0278 × 10). The estimated 1RM was used for the exercise prescription that included chest press, lat pulldown, leg press, overhead press, bicep curl, triceps extension, knee extension, and knee flexion (Hammer Strength Select machines, Life Fitness, Franklin Park, IL, USA) (Table 2). Each exercise session included a dynamic warm-up and static cool-down stretches, and all sessions (~45 minutes duration; 3× weekly) were supervised by trained personnel. Since the initial prescribed lift weight was less than the lightest weight of the machines for the participant, dumbbells, and resistance tubes with handles were employed until the participant could transfer to the machines. The intervention used progressive overload to improve strength and promote the accretion of lean body mass by gradually increasing the intensity and number of sets, thereby increasing the volume-load (Table 2). Strength measures were conducted at baseline and study weeks 4 and 8 to assess gains in strength. Anthropometric and blood pressure measurements, collection of fasting blood, and self-reported depressive symptoms assessments were conducted at the same timepoints. Height was recorded on a wall-mounted height rod (TANITA Corp, Arlington Heights, IL, USA), and body weight and composition were measured using bioelectrical impedance methods (TANITA scale, Model TBF-400, TANITA Corp, Arlington Heights, IL, USA). Blood was used for HbA1c, CRP, fasting glucose, and fasting insulin measurements (Sonora Quest Laboratories, Phoenix, AZ, USA). The homeostatic model assessment of insulin resistance (HOMA-IR) was calculated as: (fasting glucose (mg/dL) × (fasting insulin (μIU/mL))/405. Serum lipopolysaccharide-binding protein (LBP, an inflammatory marker associated with obesity-related insulin) was measured using a human enzyme-linked immunosorbent assay (ELISA) kit (HK315-02, Hycult Biotech, Uden, The Netherlands).

**Table 2:** Progression of the resistance exercise intervention by week.

Week	Sets	Repetitions	Rest	Target % 1RM*
1	1	12	30	50
2	2	12	30	55
3	2	12	30	50
4	2	12	30	60
5	2	16	60	60
6	3	10	60	65
7	3	10	60	60
8	3	10	60	70

*1RM is the maximum weight lifted for 10 reps divided by (1.0278–0.0278 × 10).

## Outcome and follow-up

The participant developed biceps tendonitis at week 5 of the intervention, and her physical therapist requested that the participant not perform chest press and overhead press. We also removed the bicep curl from her exercise prescription, and the participant continued the training protocol for the remaining five muscle groups. Following the 8-week RT training intervention, markers for strength, depressive symptomology, and fitness showed physiologically relevant improvements (Table 1). Total strength (the sum of 10 RM for lat pulldown, leg press, tricep extension, knee extension, and knee flexion) improved by 135% concomitant with a 4% increase in fat-free mass and a slight reduction in body fat percentage (−1.4%). Heart rate was reduced by nearly 14% to 70 bpm. Depression scores fell 24% from baseline values for both CES-D and POMS following the intervention suggesting a benefit of RT for moderating depressive symptomology. Considering the patient’s chronic use of antihistamine medication, we examined the fatigue and vigor sub-scores from the POMS measure, both scores improved after the 8-week intervention (−17% and +100% respectively), but these sub-scores remained substandard.

RT did not improve the traditional markers of the diabetic condition in the participant: HbA1c, fasting glucose, or HOMA-IR. RT also did not improve systemic inflammation as indicated by serum concentrations of LBP and CRP.

## Discussion

**Figure 1:**
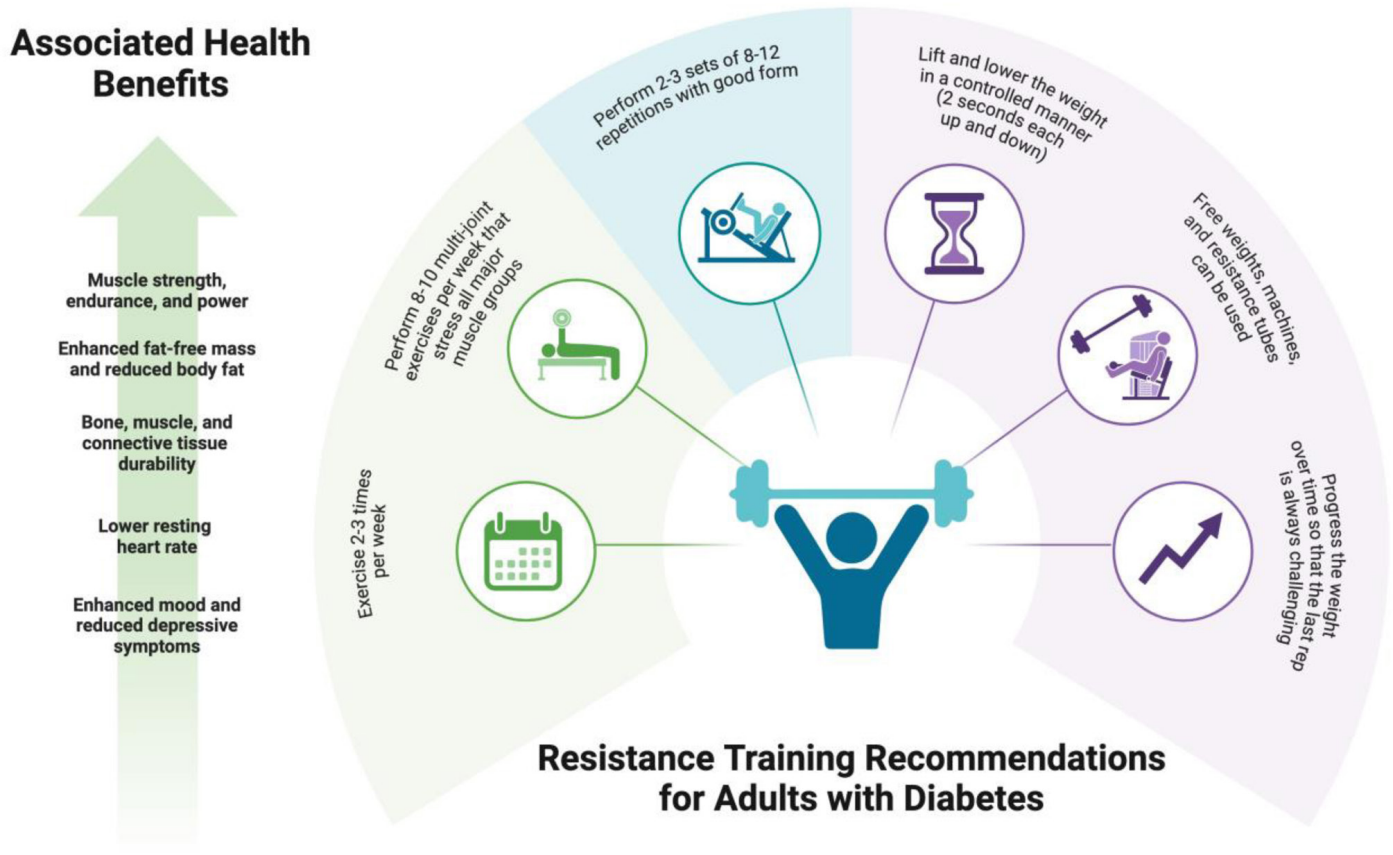
Resistance training recommendations for adults with diabetes. Adapted from Fiataraone Singh *et al.* (10).

The improvements in strength are consistent with previous studies examining the benefits of RT in individuals with diabetes (6) and have important relevance since strength is inversely linked to overall mortality in individuals with diabetes (9). However, based on normative values (1-RM) for leg press in older women, our participant fell below the 25th percentile both at baseline and at week 8, indicating the importance of maintaining RT adherence to continue gains in strength. Similar to strength data, there is a significant link between heart rate and mortality in individuals with diabetes. In the Diabetes Heart Study data set (*n* = 1315), a 1-SD rise in heart rate (~11 bpm) increased the risk of mortality by 20% (10). Hence, the importance of RT for maintaining strength and promoting cardiovascular fitness should be communicated to patients (Fig. 1).

A key aim of this case investigation was to examine the impact of supervised RT for reducing depressive symptoms. At baseline, the participant scored near or above the cut-offs for clinical depression risk on the POMS and CES-D measures respectively, and after the 8-week RT protocol, depression scores were substantially lowered to normal ranges. It is noteworthy that these improvements occurred despite the participant being treated with dulaglutide since 2022, a GLP-1 receptor agonist that has been shown in recent reports to significantly reduce depressive symptoms in individuals living with diabetes (11). Only a few trials have examined the impact of supervised RT on depression in adults with T2D: a trial conducted in Puerto Rico with 29 participants and a trial in Washington State with a small sample size (*n* = 5). Both trials did report improvements in self-reported depressive symptomology (12, 13).

Depression is a persuasive disorder among individuals with T2D and adversely impacts disease management and progression. Together these data further support an important role for RT in promoting healthful outcomes for individuals with T2D. RT, however, did not improve the traditional markers of the diabetic condition in the participant: HbA1c, fasting glucose, or HOMA-IR. Although the published data are conflicting, other investigators report similar findings. RT also did not improve systemic inflammation as indicated by LBP and CRP measurements in this participant emphasizing the need for a comprehensive treatment strategy. Simple, rapid assessments can be performed to determine the fitness and mental health of individuals with T2D, and incorporating an exercise prescription to standard care to address these key health determinants will empower many patients to actively engage in their health care.

Additionally, previous investigations have demonstrated that training under the guidance of a certified personal trainer was the best modality for an individual to achieve their fitness objectives (14). Patients can locate certified fitness trainers at professional online directories such as the American College of Sports Medicine (ACSM) ProFinder or the US Registry of Exercise Professionals (USREPS) websites.

## Declaration of interest

The authors declare that there is no conflict of interest that could be perceived as prejudicing the impartiality of the study reported.

## Funding

The research did not receive any specific grant from any funding agency in the public, commercial, or not-for-profit sector.

## Patient consent

Written informed consent for publication of their clinical details was obtained from the participant.

## Author contribution statement

MIH was the study coordinator responsible for recruitment, consenting of participant, designing and overseeing the exercise intervention, strength testing, blood collection and analyses, and data interpretation; ABD and JR (Certified Personal Trainers) were responsible for the on-site training of the participant; ED was responsible for study conception and design; CSJ senior investigator and study supervisor contributed to study conception, study design, data interpretation, and manuscript preparation. Physician permission to participate in this research was obtained by the participant.

## Patient’s perspective

At the 6-month follow-up visit, the participant expressed she would like to go back to lifting weights and exercising more often. She emphasized that meeting the personal trainer at the gym had kept her accountable and was a key motivation to finish the study, a comment that reinforces the need to incorporate exercise prescriptions in standard care to empower patients to actively engage in their health care.

## References

[1] Kant R Yadav P Barnwal S Dhiman V Abraham B & Gawande K. Prevalence and predictors of depression in type 2 diabetes mellitus. Journal of Education and Health Promotion 2021 10 352. (10.4103/jehp.jehp_1507_20) 34761038 PMC8552283

[2] Feng Z Tong WK Zhang X & Tang Z. Prevalence of depression and association with all-cause and cardiovascular mortality among individuals with type 2 diabetes: a cohort study based on NHANES 2005–2018 data. BMC Psychiatry 2023 23 490. (10.1186/s12888-023-04999-z) 37430235 PMC10331954

[3] Wium-Andersen IK Osler M Jørgensen MB Rungby J & Wium-Andersen MK. Diabetes, antidiabetic medications and risk of depression - a population-based cohort and nested case-control study. Psychoneuroendocrinology 2022 140 105715. (10.1016/j.psyneuen.2022.105715) 35338947

[4] Stanimirovic J Radovanovic J Banjac K Obradovic M Essack M Zafirovic S Gluvic Z Gojobori T & Isenovic ER. Role of C-reactive protein in diabetic inflammation. Mediators of Inflammation 2022 2022 3706508. (10.1155/2022/3706508) 35620114 PMC9129992

[5] Pollack RM Donath MY LeRoith D & Leibowitz G. Anti-inflammatory agents in the treatment of diabetes and its vascular complications. Diabetes Care 2016 39 (Supplement 2) S244–S252. (10.2337/dcS15-3015) 27440839

[6] Hsieh PL Tseng CH Tseng YJ & Yang WS. Resistance training improves muscle function and cardiometabolic risks but not quality of life in older people with type 2 diabetes mellitus: a randomized controlled trial. Journal of Geriatric Physical Therapy 2018 41 65–76. (10.1519/JPT.0000000000000107) 27893563

[7] Bay E Hagerty BM & Williams RA. Depressive symptomatology after mild-to-moderate traumatic brain injury: a comparison of three measures. Archives of Psychiatric Nursing 2007 21 2–11. (10.1016/j.apnu.2006.07.005) 17258103

[8] van Dijk SEM Adriaanse MC van der Zwaan L Bosmans JE van Marwijk HWJ van Tulder MW & Terwee CB. Measurement properties of depression questionnaires in patients with diabetes: a systematic review. Quality of Life Research 2018 27 1415–1430. (10.1007/s11136-018-1782-y) 29396653 PMC5951879

[9] Hamasaki H. What can hand grip strength tell us about type 2 diabetes?: mortality, morbidities and risk of diabetes. Expert Review of Endocrinology and Metabolism 2021 16 237–250. (10.1080/17446651.2021.1967743) 34402694

[10] Prasada S Oswalt C Yeboah P Saylor G Bowden D & Yeboah J. Heart rate is an independent predictor of all-cause mortality in individuals with type 2 diabetes: the diabetes heart study. World Journal of Diabetes 2018 9 33–39. (10.4239/wjd.v9.i1.33) 29359027 PMC5763038

[11] Pozzi M Mazhar F Peeters GGAM Vantaggiato C Nobile M Clementi E Radice S & Carnovale C. A systematic review of the antidepressant effects of glucagon-like peptide 1 (GLP-1) functional agonists: further link between metabolism and psychopathology. Journal of Affective Disorders 2019 257 S0165. (10.1016/j.jad.2019.05.044) 31153593

[12] Lincoln AK Shepherd A Johnson PL & Castaneda-Sceppa C. The impact of resistance exercise training on the mental health of older Puerto Rican adults with type 2 diabetes. Journals of Gerontology. Series B, Psychological Sciences and Social Sciences 2011 66 567–570. (10.1093/geronb/gbr034) 21571703 PMC3155029

[13] Putiri AL Lovejoy JC Gillham S Sasagawa M Bradley R & Sun GC. Psychological effects of Yi Ren Medical Qigong and progressive resistance training in adults with type 2 diabetes mellitus: a randomized controlled pilot study. Alternative Therapies in Health and Medicine 2012 18 30–34. 22516850

[14] Fiataraone Singh M Hackett D Schoenfeld B Vincent HK & Wescott W. Resistance Training for Health. Indianapolis, IN, USA: American College of Sports Medicine, 2019.

